# Factor structure and diagnostic efficiency of the Myanmar version BDI-II among substance users

**DOI:** 10.1186/s12991-019-0236-4

**Published:** 2019-07-22

**Authors:** Khine Lae Win, Norito Kawakami, Gyaw Htet Doe

**Affiliations:** 1 Department of Mental Health, Graduate School of Medicine, The University of Tokyo, 7-3-1 Hongo, Bunkyo-ku, Tokyo, 113-0033 Japan; 2 Department of Psychiatry and Mental Health, Defense Services Medical Academy, Mingaladon Township, Yangon, 11021 Myanmar

**Keywords:** Beck Depression Inventory, International Classification of Diseases, Reliability, Diagnosis efficiency, Substance users, Depression

## Abstract

**Background:**

The Beck Depression Inventory II (BDI-II) has been accepted as one of the most commonly used self-report measures for depression. However, there is no study examining the psychometric properties of the BDI-II among substance users in low- and middle-income countries such as Myanmar. Therefore, this study aimed to evaluate the suitability of using this instrument in substance users and to examine the reliability and diagnostic efficiency to be compared with the International Classification of Diseases (ICD-10) among substance users in Northern Shan State of Myanmar.

**Methods:**

A respondent-driven sampling (RDS) method was applied for recruiting the participants in this study, and total 230 substance users were recruited. On the other hand, 50 participants who visited the drop-in center (DIC) were screened for depressive symptoms using ICD-10 during the days when one consultant psychiatrist doctor was presented. These participants were interviewed face-to-face by the consultant psychiatrist using a semi-structured questionnaire including the Myanmar version of the BDI-II (mBDI-II).

**Results:**

The mBDI-II showed moderate accuracy with an area under the curve of 0.68. The optimal cutoff score was 10 with the highest Youden index (0.48), and it had high sensitivity and specificity (0.78 and 0.70). The Cronbach’s alpha coefficients for clinically depressed and non-clinically depressed substance users were 0.91 and 0.93, respectively. Confirmatory factor analysis of the mBDI-II indicated that a three-factor solution (cognitive, affective, and somatic) was the best fit for substance users.

**Conclusions:**

The mBDI-II has sound psychometric properties among substance users with moderate accuracy and range of possible cutoff scores together with sensitivity and specificity.

## Introduction

Depressive symptoms are highly prevalent among substance users and are often a comorbid condition accompanying substance-related disorder [1–3]. The 12-month prevalence of major depressive disorder among substance users was 16.1% [4], and 30 to 45% of substance users seeking treatment are affected [1]. Proper diagnosis of depression in this population is essential for treatment. However, assessing depression in this population is difficult, as the symptoms of substance use or withdrawal, such as changes in appetite and sleep pattern, concentration difficulties, and emotional changes are similar to the symptoms of depression [5, 6]. Therefore, there is a need to develop a reliable and validated screening instrument for depression that is suitable for substance users in the community setting.

Although several depression screening instruments are available, the Beck Depression Inventory II (BDI-II) [7] is one of the most commonly used and widely accepted self-report instruments for depression. In addition, the BDI-II has been tested and validated widely among both psychiatric patients, and the general population [8]. A variety of population and age groups have been studied using the BDI-II, and many studies have addressed its psychometric properties [9–13].

Moreover, the BDI-II has been used as a screening instrument for depression among low- and middle-income countries. The BDI-II was used in validation studies among adolescents in India [14, 15] and among postpartum women in Malaysia [16].

In addition, the BDI-II has been validated among substance users [17–20], most of who were tested in the USA. These studies generally reported that the BDI-II was reliable and valid among substance users [17–21]. However, it is interesting that these studies, in which factor analyses of the BDI-II were conducted, reported that a three-factor model (with factors labeled somatic, affective, and cognitive) was found to “fit” best among substance users [18–21]. There is no study examining the psychometric properties of BDI-II among substance users in low- and middle-income countries such as Myanmar.

The purpose of this study was to evaluate the suitability of using this instrument and to examine the reliability and diagnostic efficiency to be compared with the International Classification of Diseases (ICD-10) among substance users in Northern Shan State of Myanmar.

## Materials and methods

### Participants and procedures

Current substance users were recruited for this study, through their contact with a local non-government organization (NGO) providing harm reduction services in Hseni Township, Northern Shan State of Myanmar. The inclusion criteria included being aged 18 years or older, reporting using drugs (opioids, heroin, methamphetamine, etc.) within the past month, and providing informed consent to participate. Participants were recruited from people who came to a services center for accessing harm reduction services. A respondent-driven sampling (RDS) method was applied for the main evaluation study, because RDS is known as an effective data collection method for a hidden population like substance users [22]. First, 11 seeds (initial key persons) with different age groups and residential places were purposely identified from these people in Hseni Township. Each seed was given three coupons to recruit up to three other participants who he/she knew, into the study; each participants recruited by the seed was also given the same number of coupons to recruit further participants. This recruitment chain stopped at the third wave. In total, 750 coupons were distributed, and 230 coupons were returned. On the other hand, 50 participants who visited the drop-in center (DIC) were assessed by a consultant psychiatrist if they had a diagnosis of depressive disorders using ICD-10 criteria. All participants were interviewed face-to-face using a semi-structured questionnaire including the mBDI-II. Participants were provided with some snacks as an incentive, and informed consent was obtained from all.

### Variables

#### Beck Depression Inventory II (BDI-II)

The BDI-II is a 21-item self-report measure of depressive symptoms. For each item, participants are asked to select the most appropriate of four self-evaluative statements scored from “0” to “3.” Participants were asked to respond to each item based on their experiences within the past 2 weeks. Responses were summed up to yield the total score, which could range from 0 to 63, with higher scores meaning higher levels of depressive symptoms [23].

In this study, an already translated Myanmar version BDI-II (mBDI-II) was used. The translation was done by psychiatrists in Myanmar who were currently using the translated version among psychiatric outpatients as a screening instrument in Myanmar (personal communication). However, the translation process did not follow the translation, adaption, and validation of scale guidelines [24], and the translated scale had not previously been tested for reliability and validity. Before the present study, we conducted a cognitive interview for the scale among 10 substance users who were sampled ad hoc at the DIC. We found that the scale was easily completed by the participants; however, the participants gave feedback that item #21, “loss of interest in sex,” and item #10, “crying,” were difficult to answer.

#### Reference standard

The clinical interview was done based on the ICD-10 [24] for diagnosing the presence of depression and using clinical diagnostic criteria for the various depressive disorders in the F 32 category of depressive episodes (F32.0, F32.1, and F32.2), and F33 category of recurrent depressive disorders (F33.0, F33.1, and F33.2). The consultant psychiatrist who visited the DIC did the clinical interview using the ICD-10 guidelines mentioned above.

#### Substance uses

Substance use was measured by the structured questionnaire that was developed by United Nations Office on Drugs and Crime (UNODC). Participants were asked to recall the different kinds of drug(s) used within the past 1 month.

#### Demographic characteristics

A brief demographic questionnaire was used to collect information on participants’ age, sex, education, occupation, etc.

### Statistical analysis

Data were analyzed using the Statistical Package for Social Science version 21 program (SPSS 21) (IBM Corp., USA). Descriptive analysis was performed to describe the socio-demographic characteristics. Mann–Whitney *U* test was used to compare the score distribution between the groups. Sensitivity and specificity of the mBDI-II score were plotted against the ICD-10 diagnosis by the psychiatrist as a standard. The performance of the mBDI-II was analyzed using receiver operating characteristics (ROC) curves, and area under the ROC curves (AUC) with 95% confidence intervals was calculated using a nonparametric method. Sensitivity and specificity were calculated for an original cutoff score (10+) [7], a cutoff score proposed by a previous study of substance users [14–25], and the optimal screening cutoff score based on the closest point to the left hand corner, and the Youden index [25].

To examine the factor structure of the mBDI-II, confirmatory factor analysis (CFA) was done in this study. Using CFA, we compared four distinct models. Model 1 constrained all 21 items to load on a single factor (depression). Model 2 was based on the oblique two-factor solution (somatic–affective and cognitive) reported by Beck et al. [7] for BDI-II normative data from psychiatric outpatients. Model 3 was based on the oblique two-factor solution (somatic–affective and cognitive) reported by Beck et al. [7] for the BDI-II normative data from colleague students. For both Models 2 and 3, items were limited to loading only on the models as reported by Beck et al. [7], variance of each factor was fixed at 1, and the two factors were allowed to correlate. Model 4 was based on a three-factor solution (cognitive, affective, and somatic) reported by Buckley et al. [21] for substance users currently in chemical dependency treatment. Variance for each factor was fixed at 1, and the three factors were allowed to correlate.

For each of the four models, four indices were used to assess model fit. First, a Chi-square fit index was calculated to compare each given model’s covariance structure with the observed covariance matrix. A relative likelihood ratio (*X*^*2*^*/df*) (RLP) was calculated in this study. Second, root mean square error of approximation (RMSEA) adjusts for model complexity and provides a measure of predicted and observed model. Third, the Comparative Fit Index (CFI) compares the observed model fit with an independent model that assumes the latent variables are uncorrelated. The last index was the Expected Cross-validation Index (ECVI). Lower ECVI values mean better fitting of the model. The reliability of the mBDI-II was analyzed using the internal consistency coefficient and corrected inter-item correlations.

## Results

### Participants’ characteristics

The present study was conducted from September to November 2016. All 230 substance users were entered into the study. Of these, 4 users were excluded due to incompleteness of the questionnaire. The remaining 226 participants comprised the study sample, out of which 40 were diagnosed with depressive illness based on clinical criteria (based on ICD-10) and the rest (*n* = 186) were healthy.

The mBDI-II median score for the participants who had been diagnosed based on ICD-10 criteria was 20.00 (0–38), and for the others, the median score was 14.00 (0–40) (*p* = 0.003) (Table 1). The participants who had been diagnosed based on ICD-10 criteria had significantly higher mBDI-II mean scores than the comparison group (*p* = 0.003).

**Table 1:** Comparison of average scores of Myanmar version of the Beck Depression Inventory II (mBDI-II) among clinically depressed and non-clinically depressed substance users in Myanmar

	Clinically depressed (*n* = 40) median (min–max)	Non-clinically depressed (*n* = 186) median (min–max)	Mann–Whitney *U* test (*p* value)
mBDI-II	20.00 (0–38)	14.00 (0–40)	0.003

The demographic characteristics of clinically depressed substance users and non-clinically depressed substance users are reported in Table 2. The majority of the participants in both groups were male, and 53.8% of the clinically depressed patients were ethnic people; however, 62.9% of non-clinically depressed substance users were Burmese. Non-clinically depressed substance users were slightly older than the clinically depressed group, and 50% of them had never attended school. Regarding drug use history of the participants, 81.0% of the participants reported having used heroin within 30 days, and 45.6% within 24 h and 29.2% of participants reported having used amphetamine-type stimulants (ATS) within 30 days and 10.6% within 24 h). These two drugs were the most commonly used drugs.

**Table 2:** Demographic characteristics of clinically depressed and non-clinically depressed substance users in Myanmar

	Clinically depressed (*n* = 40)	Non-clinically depressed (*n* = 186)
Age (mean, SD)	36.6 (9.6)	41.9 (14.6)
Sex		
Female	2 (5.0%)	18 (9.7%)
Male	38 (95.0%)	176(94.6%)
Ethnic		
Burmese	13 (32.5%)	117 (62.9%)
Ethnic group	21 (53.8%)	52 (28.0%)
Others	5 (12.5%)	18 (9.7%)
Marital status		
Single	23 (57.5%)	56 (30.1%)
Married	13 (32.5%)	116 (62.4%)
Divorced	3 (7.5%)	3 (1.6%)
Widow	–	5 (2.7%)
Separated	–	4 (2.2%)
Education		
Never attended school	2 (5.0%)	93 (50.0%)
Primary school	12 (30.0%)	38 (20.4%)
Middle school	21 (52.5%)	26 (14.0%)
High school	3 (7.5%)	15 (8.1%)
Graduate	1 (2.5%)	28 (15.1%)
Current employment status		
Unemployed	6 (15.0%)	19 (10.2%)
Full time	4 (10.0%)	80 (43.0%)
Part time	30 (75.0%)	78 (41.9%)

Drug use history	Within 30 days	Within 24 h
Opium	33 (14.6%)	23 (10.2%)
Heroin	183 (81.0%)	103 (45.6%)
Marijuana	2 (0.9%)	1 (0.4%)
Amphetamine-type stimulant (ATS)	66 (29.2%)	24 (10.6%)
Diazepam	1 (0.4%)	1 (0.4%)
Others	7 (3.1%)	6 (2.7%)

### Diagnosis accuracy

**Fig. 1:**
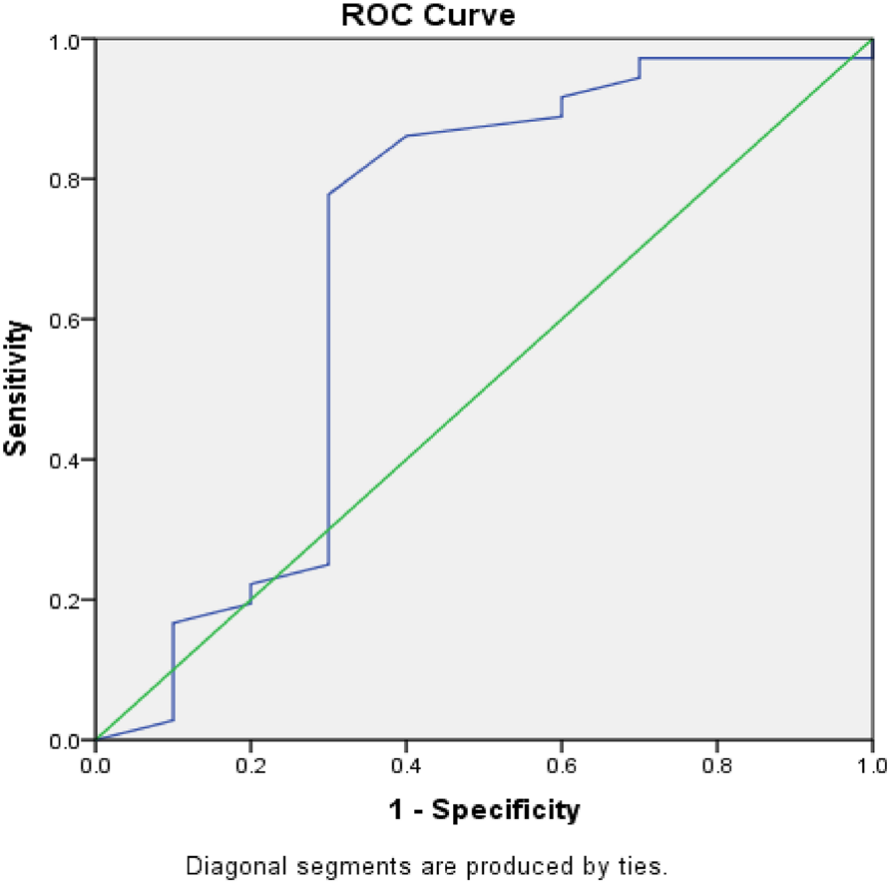
ROC curve of mBDI-II. Receiver operating characteristics (ROC) curves of the mBDI-II score for detecting clinical depression. Area under the curve was 0.69. Green line—reference line

The mBDI-II showed moderate accuracy on the AUC, and it had an AUC of 0.69 (95% CI 0.45–0.92) (Fig. 1). The optimal threshold score for screening was obtained from the ROC curves comparing the mBDI-II and ICD-10. Table 3 shows the sensitivity and specificity of cutoff scores for the detection of clinical depression with different cutoff scores from this study and other studies among substance users. According to the highest Youden index of 0.48, the optimal cutoff score was 10 with a sensitivity of 0.78 and a specificity of 0.70. The previous optimal cutoff point of 13 had a lower Youden index, sensitivity, specificity, and likelihood ratio compared to the cutoff point 10 in this study. The left-most point in the ROC curve was also 10 in this study. The likelihood ratio for positive test results (LR+) was 2.6, and likelihood ratio for negative test results (LR−) was 0.31 in this study.

**Table 3:** Sensitivity and specificity to screen clinical depression of the mBDI-II among community-living drug users in Myanmar with using the best cutoff scores in the present study and previous studies

Source	Cutoff score	Sensitivity (SE)	Specificity (SE)	Likelihood ratio (LR+, LR−)
Standard cutoff [7]	13+	0.64	0.70	2.13, 0.51
cutoff from a previous study of substance users [20]	14–25	0.95–0.73	0.36–0.75	
This study (closest to the left corner)	10+	0.78(0.07)	0.70 (0.03)	2.6, 0.31
This study (Youden index = 0.48)	10+	0.78(0.07)	0.70 (0.03)	2.6, 0.31

*SE* standard error, *LR* likelihood ratio

### Confirmatory factor analysis

Table 4 describes summary statistics for the CFA of the mBDI-II. The “goodness-of-fit” test indicated that the poorest “fit” was obtained for the one-factor solution. Of the remaining four models, the next poorest “fit” was the two-factor model. Among the remaining two models, the two-factor solution was the poorest “fit.” Therefore, the three-factor solution “fits” the best for these data.

**Table 4:** Confirmatory factor analyses

	Model 1: one-factor model	Model 2: two-factor model [7] psychiatric outpatients	Model 3: two-factor model [7] college students	Model 4: three-factor model [21] substance abusers
*X* ^*2*^	623.68	550.94	592.08	518.47
*Df*	189	188	188	186
RLP	3.29	2.93	3.15	2.79
RMSEA	0.10 (0.09–0.11)	0.09 (0.08–0.10)	0.10 (0.09–0.11)	0.09 (0.08–0.10)
CFI	0.81	0.81	0.83	0.86
ECVI	3.33	3.02	3.20	2.89
*p* value	< 0.001	< 0.001	< 0.001	< 0.001

*RLP* relative likelihood ratio *(X*^*2*^*/df)*, *RMSEA* root mean square error of approximation with 90% confidence interval, *CFI* Comparative Fit Index, *ECVI* Expected Cross-validation Index

### Internal consistency reliability and item analysis

The Cronbach’s alpha for both groups was high, and Cronbach’s alpha for the clinically depressed group was 0.91 and for the non-clinically depressed substance users was 0.93. The item-total correlation ranged from 0.15 to 0.79 for the clinically depressed sample and 0.33 to 0.76 for the non-clinically depressed substance users. Lower item-total correlations were observed for item #9, “suicidal thoughts,” #10, “crying,” and #21, “loss of interest in sex” (0.28, 0.33, and 0.15, respectively).

## Discussion

### Summary of findings

In this study, we examined the factor structure and diagnostic accuracy of the mBDI-II among substance users. CFA indicated that a three-factor solution (cognitive, affective, and somatic) was the best fit for these data. To detect depressive symptoms, which we defined the clinically depressed and the non-clinically depressed substance users, the total mBDI-II score had an area under the curve of 0.68. The optimal cutoff score was 10 for the highest Youden index (0.48) and with high sensitivity and specificity (0.78 and 0.70). An acceptable level of internal consistency reliability was obtained: The Cronbach’s alpha coefficients among clinically depressed and non-clinically depressed substance users were 0.91 and 0.93, respectively.

### Depression symptoms

The mean scores for the mBDI-II among the clinically diagnosed and the non-clinically depressed substance users were 19.9 and 14.4, respectively. This finding is similar to the mean scores in other studies among substance users ranging between 14.9 and 20.6 in the USA [26]. The mean score for the mBDI-II is also similar to the mean score for the BDI-II (13.4) in a study conducted in India, in which the study population was school adolescents [14]. The score distribution of the mBDI-II may be similar to that in other countries.

### Diagnosis accuracy

The AUC of the mBDI-II was moderate (0.68). The AUC is smaller than that reported in another study of substance users in the USA (0.82) [20]. The BDI-II may have less effective performance in screening depression among substance users in Myanmar than in high-income countries, such as the USA. In low- and middle-income countries, a smaller AUC was occasionally reported. For instance, the AUC of this study is similar to those in studies among school adolescent in India (0.66–0.67) [14, 15]. However, it should be noted that we did not diagnose depressive disorders among the non-clinically depressed sample; and thus, some of that sample may have depression. This may have resulted in underestimation of the screening performance of mBDI-II in this study.

The proposed optimal cutoff score in this study was 10 (sensitivity of 0.78 and specificity 0.70) for the diagnosis of depression among substance users. This cutoff score was less than the developer recommended cutoff score [7]. In contrast, the results for the clinically depressed substance users study [20] were between 14 and 25. Other studies of adolescents stated the cutoff score of 18 with sensitivity and specificity of 0.63 and 0.73 [15]. However, the previous studies [15, 20] did not describe the LR+ and LR−. The present LR+ 2.6 is reasonable to use, and it means that more positive test results will occur in the depressed substance users than in non-depressed users. The present LR-0.31 was close to “0,” and thus, it is less likely that the negative test result will occur in depressed substance users than in non-depressed users.

### Factor structure

A three-factor solution (cognitive, affective, and somatic) was the best fit model for these data (Table 4). Not all indices, however, indicated an acceptable fit. The RMSEA (0.09) was higher than the values of 0.05, 0.48 and 0.67 obtained in previous studies of substance users [19–21]. In addition, the CFI in this study was 0.86, while it was better (0.94) in the other study [19]. The lower fit of the solution in this study may be attributable to some of the items being less correlated with other items, as described later. The discrepancy might also be due to the study population. Our study population may be different from that in other studies in terms of the degree of substance abuse and/or treatment seeking.

### Internal consistency

The internal consistency of the mBDI-II in this study was acceptably high. This is in agreement with other studies conducted among substance users [17, 18]. A previous literature review reported that the internal consistency for the BDI-II ranged from 0.83 to 0.96 [26], which is similar to the present findings. We found a low item-total correlation of item #9 (suicidal thought), #10 (crying), and #21 (loss of interest in sex) in this study. The mBDI-II items #10 and #21 were reported in our pretest to be difficult to respond to as well. Suicide is often stigmatized more in some cultures [27]. This may be the case for substance users in Myanmar. The item on crying was reported not to converge with other depression items in some populations [28, 29]. Loss of interest in sex showed a different item functioning in a validation study of BDI-II some samples [30]. These items may be more affected by the culture of the study population and may not reflect depression appropriately.

### Limitations

Several limitations should be acknowledged for this study. Participants in this study were only substance users who identified by themselves; therefore, the result may not generalize to other populations. RDS depends on the social network of sampling population; therefore, it can limit the sample representatives. In addition, the sample size of our study is limited, and especially, the sample of participants who underwent clinical diagnosis was small. Depressive symptoms caused by substance use and withdrawal were not assessed in this study, and it can affect the validity of the instrument. An additional limitation is that we were not able to evaluate the validity of the mBDI-II by comparing responses to other depressive symptomatology instruments. Moreover, there may be clinical depressive cases in the community residents of this study and it can affect the validity of the instrument. Further studies will need to establish convergent validity and factor structure based on substance dependence severity.

## Conclusion

In conclusion, despite these limitations, this study has provided evidence that the mBDI-II has sound psychometric properties among substance users in low–middle-income countries such as Myanmar. Our study provided evidence that the diagnostic accuracy of the mBDI-II is moderate and provides a range of possible cutoff score for depression among substance users.

## Data Availability

The dataset generated and analyzed during the current study is not publicly available due to confidential assurance of the participants, however is available from the corresponding author on reasonable request.

## Abbreviations

BDI-II: Beck Depression Inventory II
ICD 10: International Classification of Diseases
NGO: non-government organization
RDS: respondent-driven sampling
DIC: drop-in center
mBDI-II: Myanmar version of the BDI-II
UNODC: United Nations Office on Drugs and Crime
ROC: receiver operating characteristics
AUC: area under the ROC curves
CFA: confirmatory factor analysis
RLP: relative likelihood ratio
RMSEA: root mean square error of approximation
CFI: Comparative Fit Index
ECVI: Expected Cross-validation Index
SD: standard deviation
ATS: amphetamine-type stimulants
CI: confidence interval
LR: likelihood ratio
